# Exploring the next “phage” of treating non-tuberculous mycobacterial infections

**DOI:** 10.1007/s15010-026-02818-7

**Published:** 2026-06-01

**Authors:** Max Kevane-Campbell, Raja Idris, Silvia Würstle, Simone Lieberknecht, Maria J. G. T. Vehreschild, Thomas A. Wichelhaus, Nils Wetzstein

**Affiliations:** 1 Department II of Internal Medicine, Infectious Diseases, Goethe University Frankfurt, University Hospital, Frankfurt am Main, Germany; 2 Mycobacterial Infection Research Unit (MIRU), Goethe University Frankfurt, Frankfurt am Main, Germany; 3 Institute for Medical Microbiology and Infection Control, Goethe University Frankfurt, University Hospital, Frankfurt am Main, Germany; 4 Molecular and Experimental Mycobacteriology, Research Center Borstel, Leibniz Lung Center, Borstel, Germany

**Keywords:** NTM, Non-tuberculous mycobacteria, Mycobacteriophages, Bacteriophages, Phages, Phage therapy

## Abstract

Non-tuberculous mycobacteria (NTM) can present a serious clinical threat due to their resistance to conventional antibiotics and their ability to cause persistent infections. As antimicrobial resistance (AMR) represents one of the most significant global health threats, mycobacteriophages (MPs)—viruses that specifically target mycobacterial species—are emerging as a promising therapeutic addition or alternative. This review explores the biological characteristics, therapeutic mechanisms, and clinical potential of MPs, with a primary focus on their use against NTM infections. Based on data from *in vitro* studies, animal models, and human compassionate-use cases, we highlight the mycobactericidal efficacy of MPs as well as their potential to complement antibiotic treatment regimens. However, there are key challenges that need to be addressed to implement MPs as therapeutics—such as limited host range, immune response interference, MP resistance, and intracellular delivery barriers. The implementation of MP banks, standardized regulatory protocols, and approved clinical trials is highly essential for MP therapy to transition smoothly into the healthcare sectors. By carrying out coordinated scientific, regulatory, and clinical efforts, MP-based therapy holds a promising potential in combating antibiotic-resistant NTM infections.

## Introduction

Mycobacteria are Gram-positive, non-motile, and non-spore-forming rod-shaped bacilli, typically measuring 0.2–0.6 μm in width and 1.0–10 μm in length. To date, more than 200 recognized mycobacterial species have been identified worldwide [1]. The members of this genus, *Mycobacterium*, exhibit remarkable diversity, with some species being highly pathogenic and capable of causing disease in both humans and animals (such as *M. tuberculosis*) and others being opportunistic pathogens [2–4].

Mycobacteria can thrive under aerobic and anaerobic conditions, though some species exhibit microaerophilic tendencies [5]. Their colony morphology varies by species, presenting as either rough or smooth, with pigmentation ranging from white, pink, and yellow to orange [5]. Pigmentation continues to be dependent on exposure to light (photochromogenic versus scotochromogenic). A defining characteristic of mycobacteria is their unique cell wall composition, which contains an exceptionally high lipid content—constituting approximately 40–60% of the bacteria’s total dry weight [6, 7]. This lipid-rich structure contributes significantly to their resilience, as well as their ability to evade antibiotic agents. Among the key glycolipids found in mycobacteria are the phenolic glycolipids of *M. leprae*, the glycopeptidolipids of the *M. avium* complex (MAC) and *M. tuberculosis*, and the acylated trehalose-containing lipooligosaccharides of *M. kansasii* and *M. tuberculosis* [8–11]. The mycobacterial cell wall serves as a formidable defence mechanism, rendering many antibiotics ineffective due to its impermeability. Structurally, the cell wall comprises three key layers: (1) an inner peptidoglycan layer, (2) a middle layer composed of highly branched arabinogalactan polysaccharides, and (3) an outer layer rich in long-chain mycolic acids [12]. Additionally, the outer membrane contains inert waxes, lipids, and loosely associated glycophospholipids [12]. The outer layer consists of polysaccharides and proteins [12], forming a carbohydrate-lipid barrier that limits permeability to hydrophilic compounds such as antibiotics and detergents. This barrier not only facilitates bacterial survival but also plays a crucial role in pathogenesis by enabling mycobacteria to evade host immune responses [12].

Non-tuberculous mycobacteria (NTM) are mycobacteria not belonging to the *M. tuberculosis* complex (MTBC), *M. leprae*, or *M. lepromatosis*. Commonly found in environmental sources such as water, soil, dust, and biofilms [13], NTM are opportunistic pathogens capable of causing a range of varying infections in humans, particularly in those with underlying health conditions (i.e. lung conditions, weakened immune systems, or individuals undergoing medical interventions) [14]. Although generally distributed and found nearly worldwide, NTM isolation and infection significantly differ based on geographic locations in terms of species incidence and their environment preferences [13]. Overall, the MAC group is most frequently isolated worldwide [15, 16]. This is also the case for most Western European countries, where MAC is followed by *M. gordonae* (mostly apathogenic) and *M. xenopi* [13, 15, 17] or the United States of America (USA), where MAC members, *M. kansasii*, and *M. abscessus* are the most frequently isolated NTM [13, 15].

Although NTM infections can mimic diseases and symptoms similar to tuberculosis, they do not generally respond to classic drug treatment regimens for *M. tuberculosis* [18]. The treatment of NTM disease is often complex and limited in therapeutic options, as the usage of drugs is commonly associated with adverse reactions and prolonged treatment durations (at times consisting of months to years) [18–21]. Even following initial treatment response, many patients can experience a relapse from incomplete treatment or possible reinfection (10–60% within 6–12 months of initial therapy completion) [18, 21–24]. Because of this, alternative treatment methods have been explored [25–28]. However, due to encouraging outcomes in individual case series, phage therapy has gained a lot of attention in recent years [29, 30].

## Overview of mycobacteriophages

Bacteriophages, or simply phages, are viruses that can specifically infect and lyse bacteria. They are among the most abundant entities on Earth, with an estimated 10³¹ particles in the biosphere—roughly a trillion phages per grain of sand globally [31–33]. These viruses exhibit a degree of host specificity, often targeting only particular bacterial species or strains [34]. While their clinical development was largely put aside in the West following the discovery and potential of antibiotics, the former Union of Soviet Socialist Republics (USSR) and its former republics (i.e. Georgia) continued to explore phage therapy for infectious disease treatment [35, 36].

Phages typically measure between 30 and 500 nm in length and are found in nearly every environment where bacteria exist [31]. Structurally, they are composed of an isometric head (capsid) that encases their genetic material—either DNA or RNA (single- or double-stranded)—and a majority of these phages possess one of three basic structural forms; isometric head with a tail, an isometric head without a tail, or a filamentous form [37–41].

**Fig. 1:**
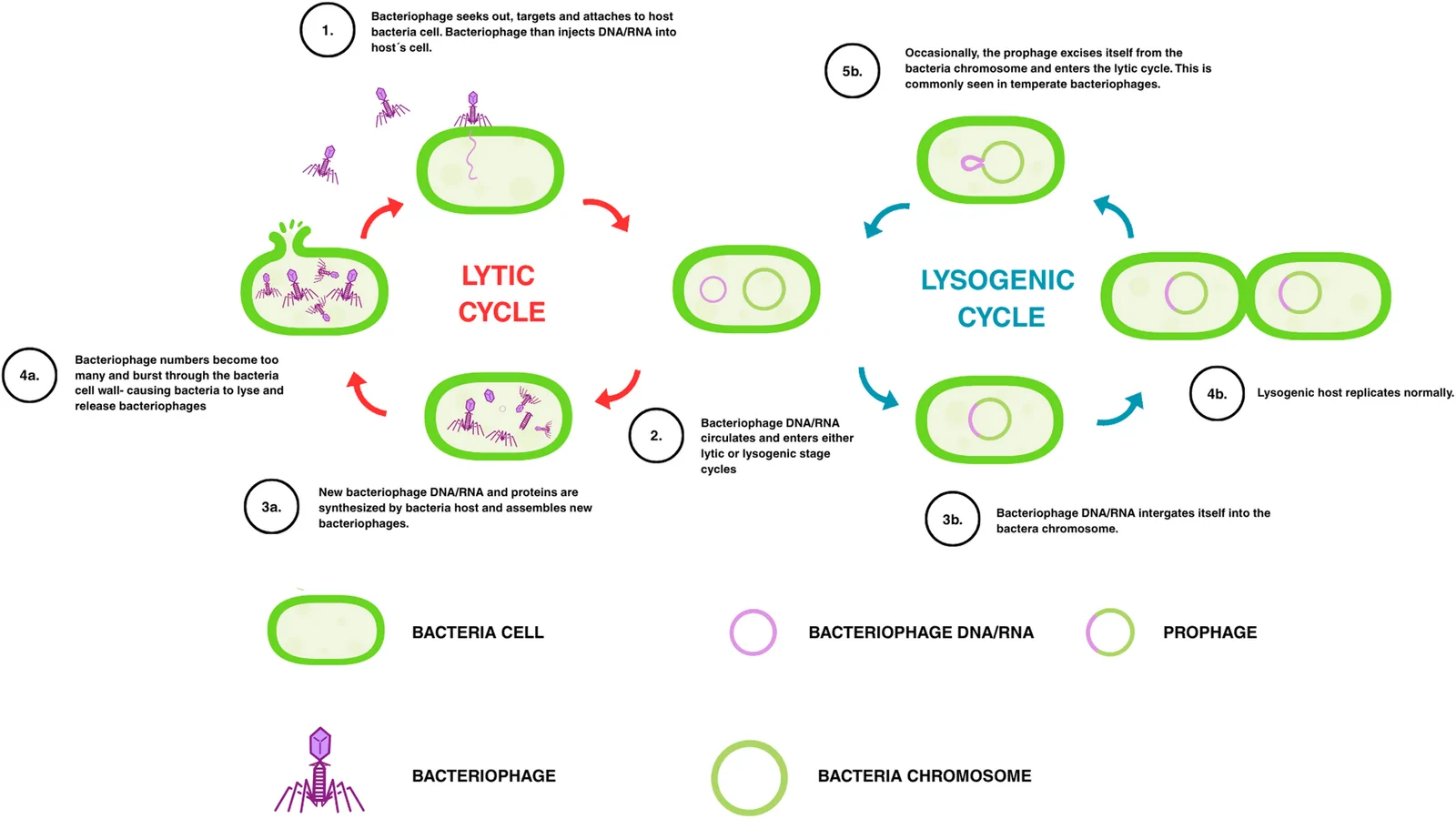
Illustration of the lysogenic and lytic cycles of bacteriophages. Figure illustration was digitally drawn using Clip Art Studio software, version 3.1.4 for Windows software.

Phages follow a variety of life cycles, with the two most well-known being the lytic and lysogenic cycles (Fig. 1). In the lytic cycle, phage DNA/RNA is injected into the host bacterial machinery after being absorbed from the host surface. The phage genome then ¨hijacks¨ the cell´s inner mechanisms and induces a shift of the host´s protein machinery to begin replicating and assembling new phage particles, eventually lysing the host and releasing on average from twelve to over hundreds of phage progeny [37, 39, 42, 43]. Conversely, in the lysogenic cycle, the phage genome integrates into the host chromosome as a prophage, remaining dormant and replicating passively with the host’s DNA during cell division [37, 39, 44, 45]. Environmental cues may later trigger a switch from lysogenic to lytic replication. Phages capable of both pathways are classified as temperate phages.

The two other more uncommon types of life cycles are the pseudo-lysogenic and chronic cycles. Within the pseudo-lysogenic cycle, after phage DNA/RNA infects the host, the phage genome does not follow through with either the lytic or lysogenic cycles, but instead stays within the host bacterium cell in an inactive form and does not integrate into the host cell´s inner machinery [37, 46]. This form of phage life cycle can be identified as being non-productive, non-destructive, non-phage-genome reproductive, non-lysogenic, and nonchronic phage infective [37, 46]. This stalled, intermediate state of the phage can stay that way until conditions allow for the phage genome to switch to either the lytic or lysogenic cycle. Finally, the chronic life cycle is defined as when a phage causes long-term infection to the host and phage progeny are continuously being generated from the host cell without causing cell lysis or are passed down asymmetrically to the replicated daughter cells [37, 47].

The term mycobacteriophage (MP) refers specifically to phages that only infect mycobacteria species. Much of the recent expansion in discovery and characterization of these phages has been facilitated by the Science Education Alliance Phage Hunters Advancing Genomics and Evolutionary Science (SEA-PHAGES) program, a collaborative undergraduate research initiative launched in 2008 by Graham Hatfull and the Howard Hughes Medical Institute [48]. Besides others [49, 50], the most notable of phage databases, the Actinobacteriophage Database, states that as of 29/04/2026, there are 15,141 MPs that have been isolated and recorded, with 2,794 of them fully sequenced [51, 52]. These phages have been identified and isolated from 26 registered strains across 10 distinct *Mycobacterium* species, including *M. smegmatis*, *M. abscessus*, *M. aichiense*, *M. aurum*, *M. avium*, *M. chelonae*, *M. neoaurum*, *M. nonchromogenicum*, *M. phlei*, and *M. tuberculosis* (Table 1) [51].

**Table 1:** Current number of isolated mycobacteriophages and according mycobacterial host species based off information gathered from the Actinobacteriophage Database as of 29/04/2026 [51].

Host *Mycobacterium* species	MPs isolated from selected *Mycobacterium* host [*n*]	Sequenced MPs [*n*]
*M. smegmatis*	15,097	2,770
*M. abscessus*	10	2
*M. avium*	9	7
*M. phlei*	7	7
*M. neoaurum*	4	3
*M. nonchromogenicum*	1	0
*M. aurum*	6	0
*M. chelonae*	2	2
*M. aichiense*	1	1
*M. tuberculosis*	1	1

**Fig. 2:**
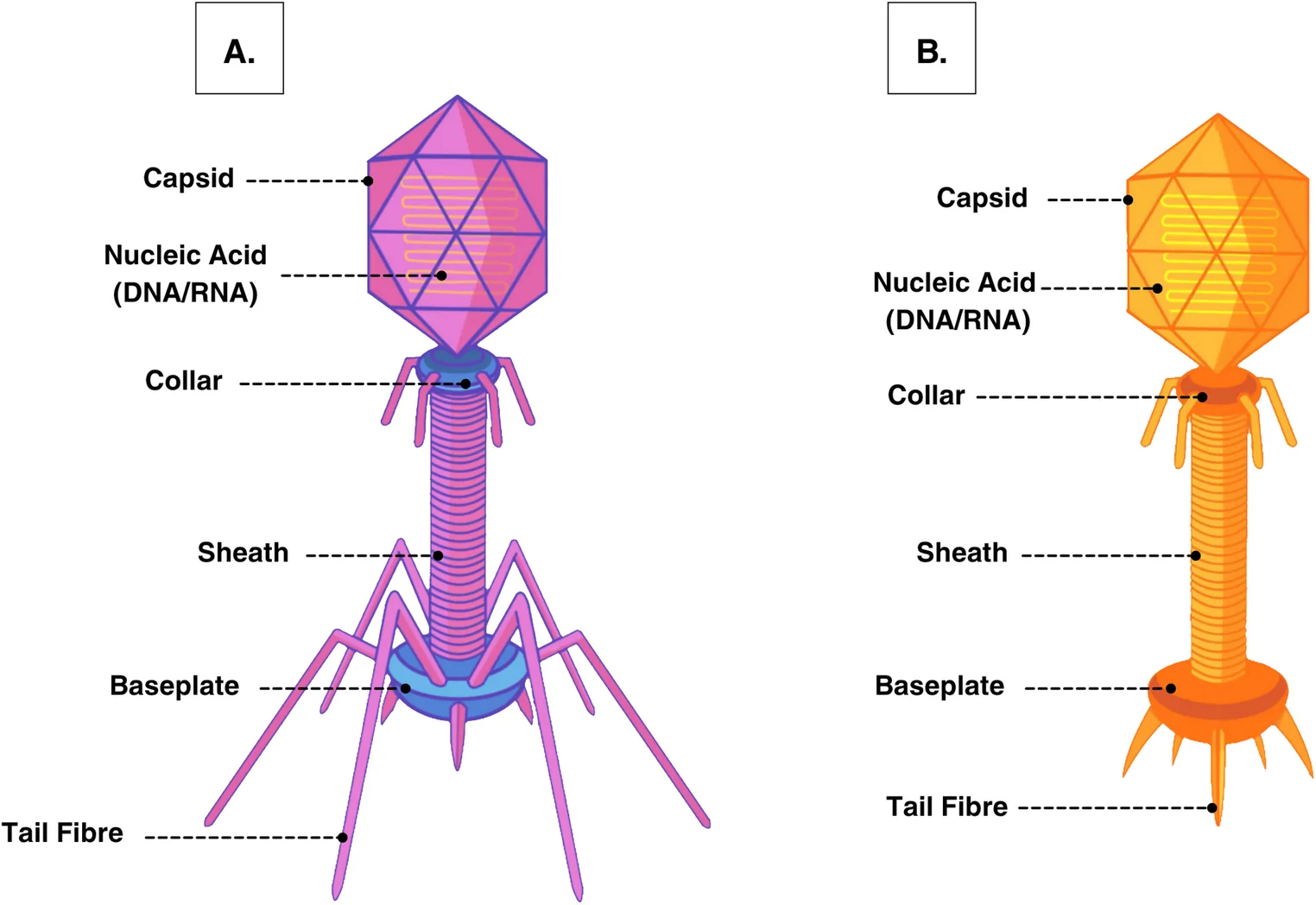
Illustrations of the two different labelled structure types of bacteriophages. **A.** Myoviral bacteriophage type structure. **B.** Siphoviral bacteriophage type structure. Of note, to date, only mycobacteriophages containing dsDNA have been identified. Figure illustration was digitally drawn using Clip Art Studio software, version 3.1.4 for Windows software.

Among these, *M. smegmatis*, particularly the mc²155 strain, is the most frequently utilized host for isolating MPs as it is a non-pathogenic, fast-growing mycobacteria species that shares many genetic and biological characteristics with other pathogenic mycobacterial species [53]. To date, the species has been used to isolate 15,097 of MP isolates [51]. All MPs characterized to date are tailed dsDNA phages—no ssDNA or RNA-based MPs have yet been identified [54]. Morphologically, they fall into two families: siphoviruses (siphoviral), with long, flexible, non-contractile tails; and myoviruses (myoviral), with contractile tails (Fig. 2). Notably, all myoviral MPs belong to a single genomic group, Cluster C—a group of MPs with an average genome length of 155,858 bp [54]. MPs can undergo either the lytic cycle or lysogenic cycle (generally in the form of temperate type phages).

The structural features of MPs vary: most have isometric capsids (40–80 nm in diameter), though some exhibit prolate heads with length-to-width ratios between 2.5:1 and 4:1 [54]. Tail lengths range from approximately 135 nm to over 350 nm [54]. Genomically, MPs display key characteristics such as: densely packed genes with little noncoding space, a modular or mosaic genome architecture, conserved synteny of virion structure genes (especially in siphoviruses), a high prevalence of small open reading frames, and many genes with unknown or predicted but unconfirmed functions [55].

## Therapeutic potential, applications, and case studies

Due to the increasing prevalence of antibiotic resistance among pathogenic mycobacteria species, there is a growing need for alternative treatment strategies [56–58]. MP-based therapy offers a promising solution, harnessing phages that specifically infect and lyse mycobacterial cells [30].

Various different kinds of *in vitro* studies have shown that MPs have the ability to lyse and reduce the concentration of more than one species of mycobacteria besides their isolation host (usually *M. smegmatis*) including species like *M. avium*, *M. avium* subspecies *paratuberculosis* (MAP), *M. tuberculosis*, *M. abscessus*, *M. ulcerans*, *M. marinum*, *M. scrofulaceum*, *M. chelonae*, and *M. fortuitum* [30, 59–66].

MPs have also been observed to be more effective against mycobacteria when combined with antibiotics in *in vitro* settings such as combining MPs with rifampicin against *M. smegmatis* [67], or combining MPs with rifabutin, imipenem, bedaquiline, clofazimine, tigecycline, amikacin, or meropenem against *M. abscessus* [64, 68, 69]. It was observed in some studies that although phage-antibiotic synergy (PAS) offered rapid reductions in NTM titre concentrations, single-dose testing and short monitoring durations likely limit sustained efficacy due to potential relapse mechanisms such as phage tolerance, incomplete killing of rough morphotypes, and sub-optimal pharmacokinetics [67, 69]. While these *in vitro* tests highlight the promising potential of combined PAS approaches, further research utilizing repeated dosing and long-term monitoring is essential to confirm durable eradication. Overall, these studies highlight that MPs can act synergistically with antibiotics and could have a place in NTM infection treatments by implementing MPs with antibiotic therapeutic regimens. However, there can also be antagonistic effects when incorporating antibiotics with phage treatment, as some antibiotics (i.e. rifampicin) have been observed to also inhibit the production of phage particles [70]. Studies using infected-animal models have helped to provide further compelling evidence supporting the efficacy MPs as a treatment against a range of mycobacterial pathogens. Positive therapeutic outcomes using MPs on animals have been observed in treating *M. tuberculosis*, *M. ulcerans*, *M. abscessus*, and *M. avium* in murine models [65, 71–73], MAP in cattle models [74], and *M. abscessus* in zebrafish models [68].

Beyond *in vitro* and animal studies, several clinical reports have demonstrated the potential of MP therapy in treating human patients suffering from mycobacterial infections (Table 2). The first documented use of phage therapy against mycobacterial infection as well as the first use of engineered phages (a term used to describe a genetically or chemically modified phages) for therapeutic purposes against mycobacteria in a human patient was seen in a compassionate case conducted by Dedrick and colleagues [75]. The case involved using phage therapy by administering a three-phage cocktail composing of lytic engineered derivatives of phages BPs and ZoeJ—both engineered to become lytic by precisely removing repressor gene 45 in ZoeJ and 33 in BPs via Bacteriophage Recombineering of Electroporated DNA (BRED)—along with MP Muddy through topical and intravenous therapy administration every 12 h for a period of at least 32 weeks following a bilateral lung transplantation to a 15-year-old patient infected with *M. abscessus* subspecies *massiliense*, who showed significant improvement in clinical response following phage administration [75]. This case was a major milestone regarding not only implementing MP-based therapy but also implementing engineered phage therapy to humans and helped to cement the potential of this antibiotic alternative when treating NTM-infections. Other positive outcomes with MP-based phage therapy—through intravenous, topical, chest wash, and nebulization administration—have been reported in a diverse range of patients, including a 26-year-old infected with *M. abscessus*, 10 patients infected with *M. abscessus,* *M. chelonae*, or MAC, and a recent case involving a 4-year-old with *M. abscessus*-based otitis (Table 2) [58, 76–79].

**Table 2:** Clinical key outcomes of patients who underwent compassionate case MP-based therapy for NTM-infections in humans.

Patient information	Age	Underlying health condition(s)	Type of infection(s)	NTM species	MP(s) used	Form(s) of administration	Clinical key outcome(s)	Reference(s)
Paediatric patient	15-year-old	Cystic fibrosis Bilateral lung transplant	Disseminated	*M. abscessus* subsp. *massiliense*	BPs∆*33*HTH_HRM10 Muddy ZoeJ∆*45*	Intravenous Topical	Reduced NTM infection Patient died due to multiple transplant complications	[58, 75, 119]
Adult patient	N/A	Scleroderma Lung transplant	Pulmonary Sternum	*M. abscessus* subsp. *massiliense*	Muddy	Intravenous Chest wash	Reduced NTM infection Patient died due to various underlying co-infections	[58, 119]
Adult patient	N/A	Cystic fibrosis	Pulmonary	*M. abscessus* subsp. *abscessus*	BPs∆*33*HTH_HRM10 Itos	Intravenous	Inconclusive Patient died due to multiple organ failure	[58, 119]
Paediatric patient	N/A	Cystic fibrosis Lung transplant	Pulmonary	*M. abscessus* subsp. *abscessus*	BPs∆*33*HTH_HRM^GD03^	Intravenous	Reduced NTM infection Patient died due to adenovirus systemic infection	[58, 119]
Adult patient	N/A	Cystic fibrosis	Pulmonary	*M. abscessus* subsp. *abscessus*	Muddy	Intravenous Nebulization	No evidential clinical benefit	[58, 119]
Adult patient	N/A	Cystic fibrosis	Pulmonary	*M. abscessus* subsp. *abscessus*	BPs∆*33*HTH_HRM^GD03^	Intravenous	No evidential clinical benefit	[58, 118]
Paediatric patient	N/A	Cystic fibrosis	Pulmonary	*M. abscessus* subsp. *abscessus*	BPs∆*33*HTH_HRM^GD03^	Intravenous	Reduced NTM infection	[58, 119]
Adult patient	N/A	Cystic fibrosis	Pulmonary	*M. abscessus* subsp. *abscessus*	Muddy	Intravenous Nebulization	Inconclusive	[58, 119]
Paediatric patient	N/A	Cystic fibrosis	Pulmonary	*M. abscessus* subsp. *abscessus*	Muddy BPs∆*33*HTH_HRM^GD03^	Intravenous Bronchoscopy	NTM infection resolved	[58, 119]
Adult patient	N/A	Cystic fibrosis Lung transplant	Pulmonary Disseminated	*M. abscessus* subsp. *abscessus*	BPs∆*33*HTH_HRM^GD03^ Itos	Intravenous	NTM infection resolved	[58, 119]
Paediatric patient	N/A	Cystic fibrosis	Pulmonary	*M. abscessus* subsp. *massiliense*	Muddy	Intravenous	Inconclusive	[58, 119]
Adult patient	81-years-old	Bronchiectasis	Pulmonary	*M. abscessus* subsp. *massiliense*	BPs∆*33*HTH_HRM10 Muddy ZoeJ∆*45*	Intravenous Nebulization	Reduced NTM infection concentration at beginning of treatment (1st month), but treatment eventually failed due to host´s phage neutralizing antibodies Treatment was switched to aerosolized phage Phage therapy was administered through terminal illness Patient died in hospice	[58, 80, 119]
Adult patient	N/A	Cystic fibrosis	Pulmonary	*M. abscessus* subsp. *abscessus*	Muddy	Intravenous Nebulization	Observed FEV1 improvement	[58, 119]
Adult patient	N/A	Cystic fibrosis	Pulmonary	*M. abscessus* subsp. *abscessus*	BPs∆*33*HTH_HRM^GD03^ D29_HRM^GD40^	Intravenous	No evidential clinical benefit	[58, 119]
Adult patient	N/A	Cystic fibrosis	Pulmonary	*M. abscessus* subsp. *abscessus*	BPs∆*33*HTH_HRM10 D29_HRM^GD40^	Intravenous	NTM infection resolved	[58, 119]
Adult patient	N/A	Arthritis	Disseminated cutaneous infection	*M. chelonae*	Muddy	Intravenous	NTM infection resolved	[58, 119]
Paediatric patient	N/A	Cystic fibrosis	Pulmonary	*M. avium* complex	Muddy	Intravenous Nebulization	Reduced NTM infection	[58, 119]
Adult patient	N/A	Cystic fibrosis	Pulmonary	*M. abscessus* subsp. *massiliense*	Muddy	Intravenous Nebulization	Inconclusive	[58, 119]
Adult patient	N/A	Cystic fibrosis	Pulmonary	*M. abscessus* subsp. *abscessus*	BPs∆*33*HTH_HRM10 Itos	Intravenous Nebulization	No evidential clinical benefit	[58, 119]
Adult patient	60 to 69-years-old	Idiopathic pulmonary fibrosis Bilateral lung transplant	Sternum	*M. abscessus* subsp. *massiliense*	Muddy BPs∆*33*HTH_HRM10^pMC09^	Intravenous	Smooth and rough colony morphotype strains were present and isolated from patient, but treatment with phages that could kill the rough strain resulted in the isolation of only the smooth strain Dual beta-lactam therapy with meropenem and ceftazidime-avibactam was later administered and provided further clinical improvement towards the patient.	[69]
Adult patient	26-years-old	Cystic fibrosis Bronchiectasis	Pulmonary	*M. abscessus* subsp. *abscessus*	BPsΔ33HTH_HRM10 D29_HRM^GD10^	Intravenous	Reduced NTM infection	[77]
Paediatric patient	4-years-old	Bilateral hearing loss Cochlear implantation	Chronic bilateral otomastoiditis	*M. abscessus* subsp. *abscessus*	Muddy BPs33 ΔHTH_HRM10 ZoeJΔ45	N/A	Reduced NTM infection Normalized inflammatory markers Resolved otorrhea Functioning cochlear implants	[79]

However, MP-based therapy is not universally successful. Dedrick and colleagues also reported that in another compassionate case, a cocktail of the same three MPs used from their previous study [75] was intravenously administered to an 81-year-old patient infected with *M. abscessus* subspecies *massiliense*-related pulmonary disease, genomically similar to the strain from the previous study [75]. An initial 10-fold reduction in bacterial sputum concentration was observed within the first month post-treatment without adverse effects, showing therapeutic promise. Despite this, bacterial levels rebounded after two months. Interestingly, bacterial isolates remained sensitive to two of the three phages even six months after administration [80]. This finding suggests that the decline in therapeutic efficacy may be attributed not to bacterial resistance (i.e. developing phage resistance), but rather to other reasons like host immune response, pharmacokinetics, not reaching the targeted site, treatment duration, dosing regimen, and/or overall phage stability as a similar treatment method was administered to a similar case with more positive outcomes [75, 80]. Of note, there seems to be higher success rates in pediatric patients for which the reasons are not entirely clear.

The potential of phages to be administered as therapeutic options is not only confined to NTM, but also to disease caused by *M. tuberculosis*. Although no case studies have been recorded using phage therapy on patients with tuberculosis, multiple *in vitro* and *in vivo* studies have tested MPs as therapeutic candidates for multidrug-resistant (MDR) and extensively drug-resistant (XDR) strains. The *in vitro* study conducted by Guerrero-Bustamante et al. observed that using a cocktail of five MPs—AdephagiaΔ*41*Δ*43*, D29, FionnbharthΔ*45*Δ*47*, Fred313_cpmΔ*33*, and Muddy_HRM^N0157^-2—can minimize the emergence of phage resistance, as well as efficiently kill various tested *M. tuberculosis* strains [81]. The study also observed that tested phages could function without antagonizing antibiotic effectiveness and infect both isoniazid-resistant and -sensitive *M. tuberculosis* strains [81]. Phages D29 and DS6A are also able to efficiently lyse *M. tuberculosis in vitro* [65]. Of key note, DS6A has been observed to efficiently reduced the *M. tuberculosis* load in humanized mice organs when administered intravenously [65] and decreased lesions in the spleens of guinea pigs [82]. Overall, these experimental data might point towards a future application of MP therapy (e.g. as part of a synergistic combinational therapy) in the fight against MDR tuberculosis.

## Challenges and limitations

While there is growing evidence supporting the use of MPs as effective therapeutic agents against various pathogenic mycobacteria, there are still several limitations that need to be addressed. A major concern is that MP therapy remains largely experimental, with limited public awareness and clinical integration to date.

One of the key risks involved in using phage therapy is the host’s physiological response to possible bacterial cell residue-based impurities (endotoxins) within purified phage solutions when administration does not adhere to general and standard guidelines. MP solutions must be highly purified and free of endotoxins for them to be clinically safe for therapeutic administration in potential patients, as impure MP solutions could result in patients developing severe complications such as inflammation, septic shock, and sepsis [83, 84]. Immune responses may also neutralize phages via anti-phage antibodies [58, 80, 85]. The previously stated study conducted by Dedrick et al. reported an 81-year-old *M. abscessus*-infected patient who developed high IgG and IgM mediated phage neutralizing antibodies as well as high IgA titres [80]. In another instance, eight patients developed phage neutralizing antibodies after post-IV MP treatment, with mixed outcomes; some had pre-existing antibodies from environmental exposure [58]. However, Nick et al. observed that a cystic fibrosis patient with pre-existing neutralizing antibodies was able to successfully receive engineered MPs and underwent a lung transplant, indicating that such antibodies may not always prevent effective treatment [77].

Another key challenge for phage therapy is the development of phage resistance by targeted bacteria. This can occur via mechanisms such as spontaneous mutations, restriction modification systems, and adaptive immunity via the CRISPR-Cas system [86]. On a positive note, phage resistant bacteria can become less virulent or more antibiotic-susceptible, likely due to examples such as altered metabolism or modified phage/antibiotic receptors [87, 88]. The study by Dedrick et al. found that rough *M. abscessus* colonies were more phage-susceptible (up to 80%), linked to mutations in *mps1* and *mps2*, while smooth colonies with mutations in polyketide synthesis, *rpoZ*, and *uvrD2* were more resistant [89]. Other examples of NTM developing phage resistance can be seen in *M. smegmatis* by how the species can deploy strategies like restriction systems and/or prophage-mediated defence [90]. However, phages have been noted to overcome these defences [91]. Strategies to combat resistance include administering phages sequentially—based on susceptibility and expected trade-offs, phage cocktails targeting diverse receptors [92–94], engineered phages attacking virulence factors [84], or combining phages with antibiotics [95].

The intracellular lifestyle of NTM, like *M. abscessus* and MAP poses another barrier, as these bacteria are shielded from extracellular phages [29, 96, 97]. However, some phages can cross epithelial barriers via transcytosis [98, 99], and both natural and engineered phages have shown to induce endocytosis, enabling them to gain entry into mammalian cells and targeting intracellular mycobacteria [100–102]. Further, the narrow host range of some MPs further complicates therapy, necessitating personalized phage matching and/or isolation. Research is also needed and currently being carried out on MP stability—including temperature, pH, and salinity sensitivity, as well as encapsulation, lyophilization, and delivery methods [60, 103–107].

**Fig. 3:**
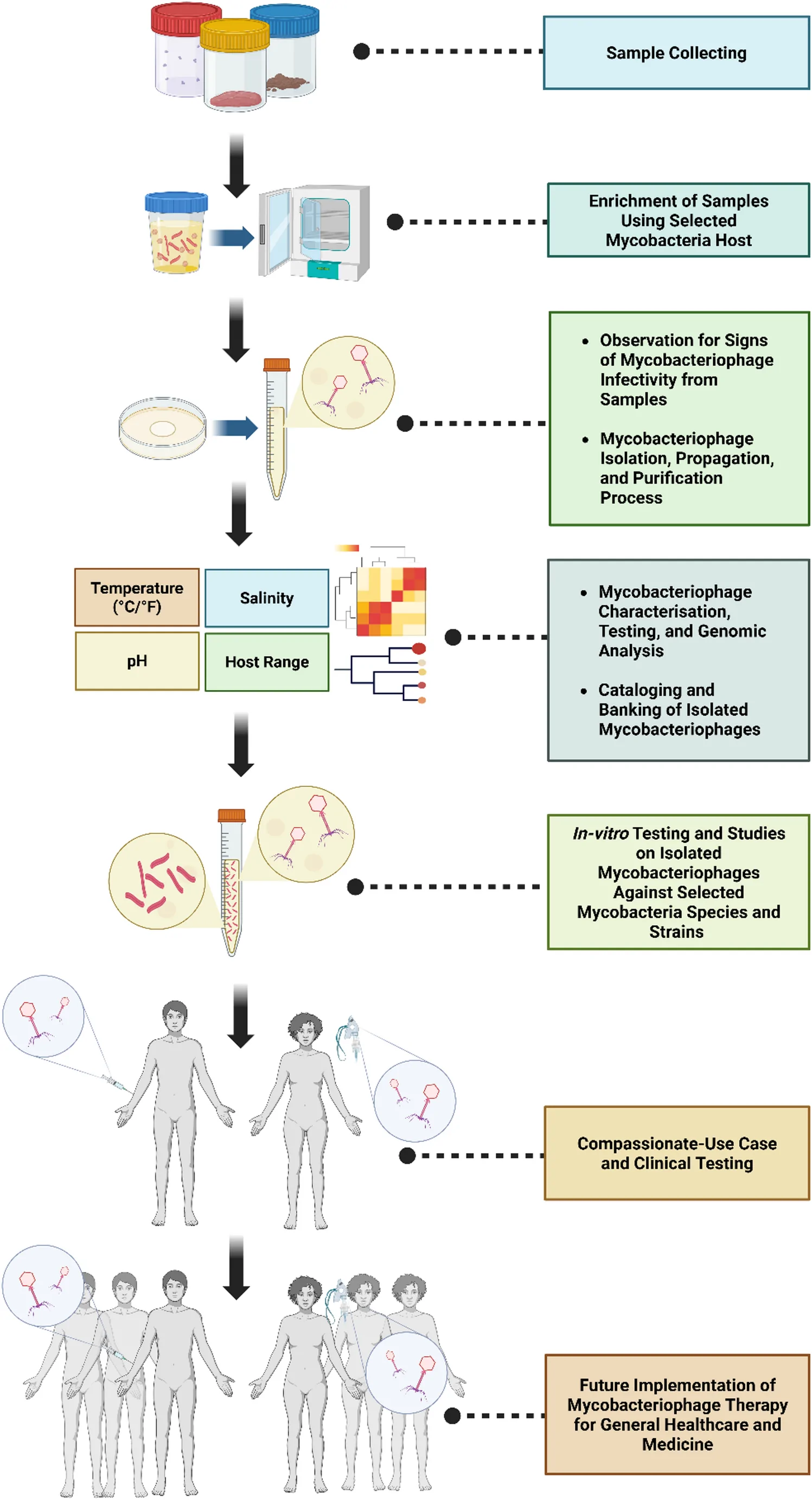
Illustration of a roadmap for the development and integration of MP therapy into modern healthcare and medicine. A stepwise layout outlining the essential steps for advancing MP therapy—from phage isolation and characterization to clinical implementation—including *in vitro* and *in vivo* validation, data registration, and integration into existing healthcare protocols. Image was created with BioRender.com

Finally, regulatory concerns must be addressed through ethical approvals, expanding public awareness, safety evaluations, and conducting rigorous preclinical and clinical studies. Identifying and overcoming these barriers is essential to validate MP therapy and ensure its clinical success against NTM infections (Fig. 3). Ethical issues of equal access and compassionate use are another hurdle when establishing access towards global phage banks. Currently, phage therapy is generally only administered on a personalised and compassionate basis. Therefore, patients residing in countries which lack healthcare centres specialized in phage therapy may face delays or denial of phage therapy treatment. The lack of large-scale, randomized controlled trials (RCTs) can create ethical concerns regarding the use of non-good manufacturing practices (GMP) phages, even when used for compassionate cases [108]. GMP compliance of large-scale production and purification of phages must be correctly incorporated to ensure that phage lysates are purified to remove any bacterial remnants—like that of endotoxins—as these can cause severe adverse reactions to patients [108]. Additionally, limited laboratory infrastructure—especially in low-resource settings—might inhibit the ability of isolating and characterising local phages [108, 109]. Public awareness, as well as engagement, could be achieved by setting up additional early-stage research programs (i.e. the SEA-PHAGES program) and expanding science communication of phage therapy on different platforms (i.e. using social media, talks, blogs, and podcasts) [110, 111].

## Future directions and clinical potential

To advance MP therapy into standard medical practice, several steps are needed. First, the development of clinical trials is essential for validating the safety and therapeutic potential of phage-based therapy. However, it is of note that as phages are uniquely different to standard antibiotics, the administration of phage therapy is a very individualised process and the possibility of a clinical trial for each phage/indication is not entirely feasible. Due to this, the administration of personalised phage therapy for infected patients is inevitable. Tested and recorded clinical data will not only help to inform healthcare guidelines but also help to foster regulatory approval. In correlation, the establishment of well-organized and catalogued phage banks are also vital. To achieve this, key steps must be implemented, from the screening and isolation of phages (lytic phages being a main priority) to finally implementing structured clinical trials of specific phage(s). Alternative trial designs might help tailor individualized treatments for patients by implementing customized phage therapy as well as personalised phage cocktails [112], PAS testing of clinical isolates and administration [113, 114], using engineered phages and phage derivatives, as well as testing the therapeutic potential of phages against bacterial isolates using automated bioinformatics pipeline tools (i.e. PhageCompass, Sphae, PhaBOX, PhageTB) and ensemble methods [114–117] (Table 2; Fig. 3). Standardized GMP phages might be used in classical trial designs such as a RCTs.

Moreover, for phage therapy to become a practical option, it must be effectively integrated with other current treatment protocols, potentially working synergistically with antibiotics when treating resistant infections. Finally, phage therapy needs to be accessible as well as the implementation of public education initiatives to help build awareness and understanding of implementing this therapeutic treatment must be incorporated. By implementing these steps, the foundation of responsible and widespread use of phage therapy might be laid on a global scale.

## Conclusion

MP-based therapy represents an exciting, compelling, and urgently needed alternative to conventional antibiotic treatments for NTM-infections. As AMR continues to rise as well as the complexity of treating NTM infections, the precision, self-replicating nature, and specificity of MPs offer a promising and novel therapeutic avenue capable of targeting drug-resistant mycobacteria without disrupting the surrounding microbiota of the host.

Evidence gathered from *in vitro *experiments, animal studies, and a growing number of individual treatment using MPs have clearly highlighted the efficacy and safety potential of MPs as a therapeutic. However, there are several scientific, clinical, and regulatory barriers that must be overcome to bring MP-based therapy into the healthcare sectors. These include addressing host immune responses, improving delivery methods for intracellular NTM, and developing reliable strategies for phage formulation and storage.

Through interdisciplinary collaboration, established research efforts, and robust support from international regulatory bodies, MP-based therapeutics might have the potential to change the way healthcare professionals treat NTM infections.

## Data Availability

No datasets were generated or analysed during the current study.
